# High-Dose Enalapril Treatment Reverses Myocardial Fibrosis in Experimental Uremic Cardiomyopathy

**DOI:** 10.1371/journal.pone.0015287

**Published:** 2011-01-27

**Authors:** Karin Tyralla, Marcin Adamczak, Kerstin Benz, Valentina Campean, Marie-Luise Gross, Karl F. Hilgers, Eberhard Ritz, Kerstin Amann

**Affiliations:** 1 Department of Pathology, University of Erlangen-Nürnberg, Erlangen, Germany; 2 Department of Pathology, University of Heidelberg, Heidelberg, Germany; 3 Department of Nephrology, Endocrinology and Metabolic Diseases, Silesian University School of Medicine, Katowice, Poland; 4 Department of Internal Medicine-Nephrology, University of Erlangen-Nürnberg, Erlangen, Germany; 5 Department of Internal Medicine, University of Heidelberg, Heidelberg, Germany; University of Bristol, United Kingdom

## Abstract

**Aims:**

Patients with renal failure develop cardiovascular alterations which contribute to the higher rate of cardiac death. Blockade of the renin angiotensin system ameliorates the development of such changes. It is unclear, however, to what extent ACE-inhibitors can also reverse existing cardiovascular alterations. Therefore, we investigated the effect of high dose enalapril treatment on these alterations.

**Methods:**

Male Sprague Dawley rats underwent subtotal nephrectomy (SNX, n = 34) or sham operation (sham, n = 39). Eight weeks after surgery, rats were sacrificed or allocated to treatment with either high-dose enalapril, combination of furosemide/dihydralazine or solvent for 4 weeks. Heart and aorta were evaluated using morphometry, stereological techniques and TaqMan PCR.

**Results:**

After 8 and 12 weeks systolic blood pressure, albumin excretion, and left ventricular weight were significantly higher in untreated SNX compared to sham. Twelve weeks after SNX a significantly higher volume density of cardiac interstitial tissue (2.57±0.43% in SNX vs 1.50±0.43% in sham, p<0.05) and a significantly lower capillary length density (4532±355 mm/mm^3^ in SNX vs 5023±624 mm/mm^3^ in sham, p<0.05) were found. Treatment of SNX with enalapril from week 8–12 significantly improved myocardial fibrosis (1.63±0.25%, p<0.05), but not capillary reduction (3908±486 mm/mm^3^) or increased intercapillary distance. In contrast, alternative antihypertensive treatment showed no such effect. Significantly increased media thickness together with decreased vascular smooth muscles cell number and a disarray of elastic fibres were found in the aorta of SNX animals compared to sham. Both antihypertensive treatments failed to cause complete regression of these alterations.

**Conclusions:**

The study indicates that high dose ACE-I treatment causes partial, but not complete, reversal of cardiovascular changes in SNX.

## Introduction

The development of left ventricular hypertrophy (LVH) and structural abnormalities of the heart and vessels is a key abnormality in chronic kidney disease (CKD) that potentially contributes to the high rate of cardiac death in this population [1]. Among the myocardial changes that accompany LVH in experimental renal failure as well as in patients with CKD the following play major roles: myocardial fibrosis [2], [3], loss of cardiomyocytes [4], thickening of intramyocardial arterioles [5]–[6] and finally marked capillary deficit causing a mismatch between cardiomyocyte hypertrophy and capillary density [7]–[8].

Recent clinical and experimental studies document that the pathogenesis of these cardiovascular abnormalities is complex. Certainly, these abnormalities are not fully explained by increased pre- or afterload or by anemia [9]–[12]. Amongst others, the local renin aldosteron angiotensin system (RAS) seems to play a decisive role [1], [13]. Other studies documented elevated angiotensin II and renin mRNA expression in the myocardium of subtotally nephrectomized animals (SNX) with moderate chronic renal failure [14], [15]. In experimental renal failure blocking the RAS with an angiotensin converting enzyme (ACE) inhibitor (ACE-I) prevented development and progression of LVH and associated structural alterations such as myocardial fibrosis and loss of cardiomyocytes [16]. In patients with CKD evidence of regression of LVH after long-term treatment with either ACE-I or combination treatment was found [17], [18]. Regression of LVH was also seen in hemodialysed patients with a policy of negative sodium balance, thus lowering blood pressure in the absence of any medication [19], [20]. These studies in human beings could not address the issue how structural alterations of the heart in CKD were affected by either ACE-I or blood pressure lowering, respectively.

Whether in experimental renal failure ACE-I can also regress prevalent cardiac abnormalities and how these were affected in detail has not been investigated so far.

These considerations prompted the present study in subtotally nephrectomized rats which had developed major cardiovascular pathology. It was particularly designed to investigate the hypothesis that high-dose treatment with the ACE-I enalapril, but not treatment with alternative blood pressure lowering drugs reversed such existing cardiovascular pathology, i.e. LVH, interstitial myocardial fibrosis, reduced myocardial capillary supply, intramyocardial arteriolar and aortic wall thickening. In a standard model of moderate experimental renal failure [21] we assessed structural changes of the heart and the aorta in untreated SNX animals at 8 weeks and in addition compared at 12 weeks untreated SNX with SNX that had been treated for 4 weeks (week 8–12) with high-dose enalapril. To exclude confounding by lowering of blood pressure we studied in parallel SNX and sham operated animals treated with the non-specific antihypertensive combination furosemide and dihydralazine.

## Materials and Methods

### 1. Animals and study design (fig. 1A)

Three months old male Sprague Dawley rats (Charles River Co, Sulzfeld, Germany), mean body weight 379±27 g, were housed at constant room temperature (21°C) and humidity (75%) and exposed to a 12 h light on, 12 h light off cycle. The animals had free access to water and were fed pellets (23.4% protein, 4.5% fat, 6% fiber, 0.4% sodium; Altromin GmbH, Lage, Germany). After a 7 days adaptation period, rats were randomly allotted to subtotal nephrectomy (SNX, n = 34) or sham operation (sham, n = 39). As described before [16] rats were subtotally nephrectomized in two steps: first, the right kidney was surgically removed and kidney weight was carefully protocolled, then, one week later weight controlled removal of cortical tissue of the hypertrophied left kidney corresponding to 2/3 of the weight of the right kidney. This standardized procedure of two-step, weight controlled surgical resection of renal cortex resulted in a very moderate and stable degree of renal failure with a minor increase in systolic blood pressure, if any. Using the above procedure of moderate two-step subtotal nephrectomy the total nephron number is reduced from approximately 60,000 to 15,000.

**Figure 1:**
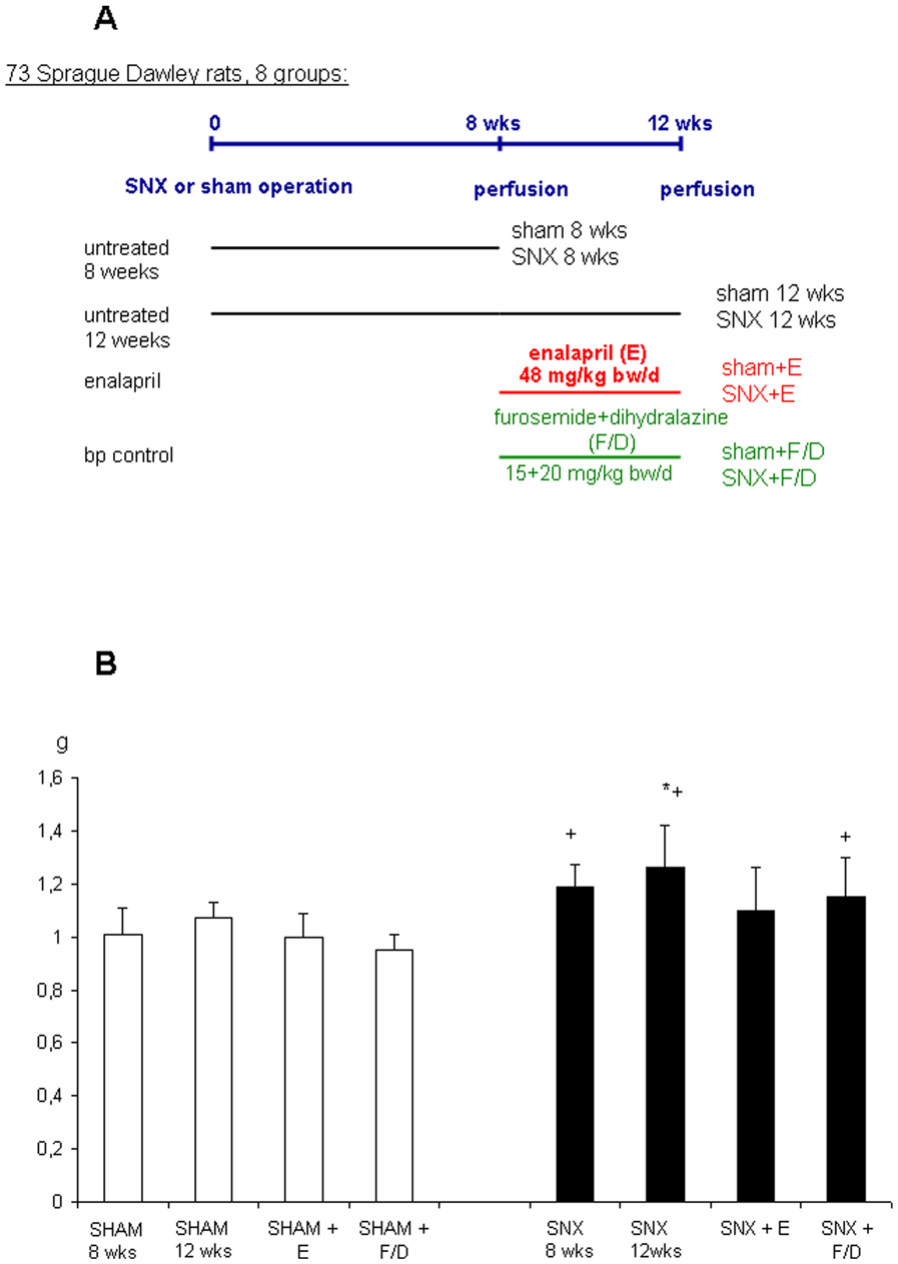
Experimental protocol (A) and left ventricular weight (B). A. Experimental protocol. B. Effect of treatment with the ACE-I enalapril or furosemide/dihydralazine on left ventricular weight (g). The increase in left ventricular weight (g) in untreated SNX at week 12 is completely prevented by enalapril, not by furosemide/dihydralazine treatment.

Eight weeks after the second operation, one group of sham and SNX animals was sacrified in order to clearly demonstrate the findings before the onset of therapy (sham 8 wks, SNX 8 wks). The remaining sham and SNX rats were randomly allotted to 2 treatment arms for another 4 weeks (fig. 1A): (i) enalapril treatment (E, 48 mg/kg bw per day, sham+E, SNX+E), (ii) furosemide (F) + dihydralazine (D) treatment (F/D, 15+20 mg/kg bw, sham+F/D, SNXF/D). One group of sham and SNX animals was left untreated (sham 12 wks, SNX 12 wks). Treatment was given by adding the drugs to the drinking water at concentrations calculated to deliver the above mentioned dose. Daily food and water consumption were monitored and the doses were adjusted. The enalapril dose used in the current study exceeds the antihypertensive dose used in previous prevention studies by a factor of 4. In previous studies our group had shown that treatment with the ACE-I ramipril prevented the development of LVH and myocardial fibrosis in SNX rats [4], [16]. When designing the present study we reasoned that in contrast to prevention [16], regression of already altered heart morphology might require a higher dose of E, e.g. 48 mg/kg body weight [22] similar to the high doses necessary to cause regression of glomerular sclerosis [23]. In the absence of studies on regression of cardiovascular alterations we chose the dose used by Ikoma et al. [22] who showed that in SNX a dose of 48 mg/kg bw enalapril, but not lower doses caused regression of glomerular lesions. The doses of F and D were chosen according to a previous study^5^ and adjusted to induce a comparable blood pressure lowering.

#### Ethics statement

All animal work has been conducted according to relevant national and international guidelines. Formal approval was given by the local authorities (Regierungspräsidium Karlsruhe, AZ 35-9185.81/69/98).

### 2. Blood pressure (bp) and urinary albumin measurements

Systolic bp and heart rate were measured at weeks 2, 5, 7, 9 and 11 using tail plethysmography in conscious rats that were acquainted to the measuring conditions. In each animal 6 consecutive measurements per session were performed.

Seven and 11 weeks after SNX animals were placed in metabolic cages and 24 h urine was collected to measure urine volume, electrolyte and albumin excretion [24].

### 3. Tissue preparation, morphometry and stereology

After the above mentioned recordings and blood sampling the experiment was terminated by retrograde perfusion fixation via the abdominal aorta Perfusion pressure was adapted according to the in vivo blood pressure of the animals, i.e. 120–140 mmHg. Perfusion was started with rheomacrodex/procainhydrochloride in order to prevent interstitial edema and artifacts due to various states of vasodilation, followed by either glutaraldehyde for morphometric and stereological investigations or icecold NaCl for molecular studies [5]. After the perfusion, the heart of each animal was taken out and the total heart weight aswell as the left ventricular weight were determined. From glutaraldehyde fixed hearts tissue samples and sections were obtained and stained according to the orientator method (for details see [16], [25]). Thus, semithin sections of 8 random samples of the left ventricular muscle including the septum were cut and examined by light microscopy with oil immersion and phase contrast at a magnification of 1∶1000. All investigations were performed in a blinded manner, i.e. the observer was unaware of the study group the animal belonged to. Volume density (V_V_) of capillaries, interstitial tissue and myocytes was obtained using the point counting method according to the equation P_P_  =  V_V_ (with P_P_ is point density). Reference volume was the total myocardial tissue (exclusive of non-capillary vessels, i.e. arterioles and veins, and tissue clefts). Vascular geometry of intramyocardial arterioles, i.e. vessels with lumen diameters between 20 and 120 µm and at least one muscular layer, was analysed using planimetry and a semiautomatic image analysis system (Analysis, SIS, Münster, Germany) as described in detail [5], [6]. Thereby, mean wall thickness, lumen diameter, media and lumen area were determined in every arteriole that was present in all semithin sections per animal.

A 3 mm thick slice of the descending aorta was also embedded in Epon Araldite and semithin sections were prepared for quantitative and qualitative evaluation of the aortic wall. The remaining cardiac and aortic tissue was embedded in paraffin and 3 µm thick sections were prepared and stained with HE and Sirius red (for visualization and quantification of fibrous tissue).

### 4. TaqMan PCR for cardiac TGF-β, TIMP-1 and TMP-2 gene expression

Total RNA was extracted with the Qiagen MiniKit (Qiagen GmbH, Hilden, Germany). First-strand cDNA was synthesized with TaqMan reverse transcription reagents (Applied Biosystems, Darmstadt, Germany) using random hexamers as primers. Reactions without Multiscribe reverse transcriptase were used as negative controls for genomic DNA contamination. PCR was performed with a Step One Plus Sequence Detector System FastSYBR Green Universal PCR Master Mix (Applied Biosystems), as described previously [26]. All samples were run in triplicate. Specific mRNA levels in hypertensive animals relative to UNX controls were calculated and normalized to a housekeeping gene with the Δ-Δ-C_T_ method as specified by the manufacturer (http://www3.appliedbiosystems.com/cms/groups/mcb_support/documents/generaldocuments/cms_040980.pdf).

Primer pairs and probes for transforming growth factor- β (TGF-β) [27] and tissue inhibitor of metalloproteases-1 and -2 (TIMP-1) were designed using Primer Express software (Perkin Elmer, Foster City, CA, USA) [28]. The relative amount of the specific mRNA was normalized with respect to 18S rRNA. All samples were run in triplicate.

### 5. Statistics

All statistical analysis was performed with SPSS 13. Data are given as mean ± standard deviation apart from the results of the TaqMan PCR which are provided as box plots. ANOVA was used for comparison of means followed by appropriate post-hoc tests. If distributional assumptions were in doubt the nonparametric Kruskal-Wallis-test was chosen. The zero-hypothesis was rejected at p<0.05.

## Results

### 1. Animal data

#### Enalapril (E) had no effect on body weight, but reversed left ventricular hypertrophy (LVH) in SNX animals (table 1, fig. 1B)

At the end of the present experiment body weight was not significantly different between the groups. Eight and 12 weeks after SNX the weight of the left ventricle (LVW) was significantly higher in untreated SNX and SNX+F/D compared to sham indicating left ventricular hypertrophy (LVH). After 12 weeks LVW was significantly lower in SNX+E compared to untreated SNX documenting regression of LVH in SNX animals (fig. 1B). S-creatinine and urea as well as albuminuria were significantly higher in all SNX groups compared to sham (table 1). Of note, neither enalapril (E) nor furosemide/dihydralazine (F/D) treatment significantly lowered these parameters.

**Table 1:** Animal data: Effect of treatment with enalapril (E) or furosemide/dihydralazine (F/D) from week 8–12 in sham-op and SNX rats, respectively.

group	body weight [g]	S-creatinine [mg/dl]	S-urea [mg/dl]	albuminuria [mg/day]
**SHAM 8 wks** **n = 10**	500±20	0.48±0.08	40.8±2.9	2.76±2.65
**SHAM 12 wks** **n = 10**	552±24	0.49±0.06	44.2±3.3	4.38±4.90
**SHAM +E** **n = 11**	538±28	0.53±0.08	56.1±11.8	0.81±0.63
**SHAM + F/D** **n = 8**	586±39	0.4±0.05	42.4±5.26	1.31±1.34
**SNX 8 wks** **n = 8**	486±31	0.85±0.19 **a**	86.5±13.9 **a**	115±116 **a**
**SNX 12 wks** **n = 8**	531±52	0.88±0.23 **a**	97.9±32.1 **a**	277±148 **a**
**SNX +E** **n = 11**	491±25	0.92±0.08 **a**	107±19 **a**	258±220 **a**
**SNX + F/D** **n = 7**	544±19	1.01±0.57 **a**	118.1±67 **a**	192±195 **a**
**analysis of variance**	ns	p<0.001	p<0.05	p<0.001

mean ± standard deviation, ^1^ weight after perfusion fixation.

a) p<0.05 vs. corresponding SHAM.

b) p<0.05 vs. SNX 8 wks.

c) p<0.05 vs. SNX 12 wks.

d) p<0.05 vs. SNX+E.

#### Comparable effects of enalapril (E) and furosemide/dihydralazine (F/D) on systolic blood pressure (bp) (fig. 2A)

Two weeks after SNX systolic bp was not significantly different between the groups. From week 5 onward bp was moderately, but significantly higher in untreated SNX than in untreated sham. Treatment with E and F/D significantly and comparably lowered bp in SNX and sham compared to untreated animals. Of note, at week 7, i.e. 1 week before the initiation of antihypertensive treatment bp was highest in the SNX+F/D group and this might have some potential effect on any of the readout parameters although this remains speculative.

**Figure 2:**
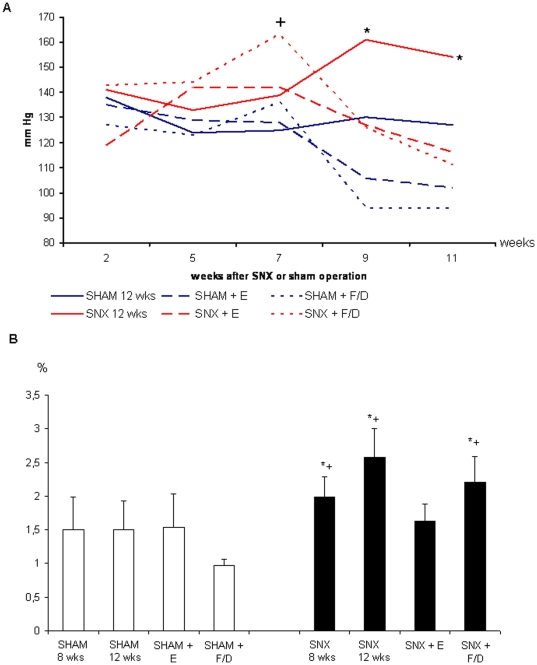
Effect of treatment with the ACE-I enalapril or furosemide/dihydralazine on systolic blood pressure (A) and myocardial interstitial fibrosis (B). **A.** Enalapril (E) and furosemide/dihydralazine (F/D) treatment lowered systolic blood pressure (bp) in sham and SNX to the same extent. Two weeks after SNX systolic bp was not significantly different between the groups. From week 5 onward bp was significantly higher (p<0.01) in untreated SNX than in untreated sham. At week 7 bp was highest in the SNX+F/D group. Treatment with E and F/D significantly and comparably lowered bp in SNX and sham compared to untreated animals. Mean of systolic blood pressure measurements at weeks 2, 5, 7, 9 and 11 using tail plethysmography in conscious rats that were acquainted to the measuring conditions. *: p<0.01 compared to all other groups. +: p<0.05 compared to all other groups. **B.** The increase in myocardial interstitial tissue (%) in untreated SNX at week 12 is completely prevented by enalapril, but not by furosemide/dihydralazine treatment. *: p<0.05 vs SNX 12 weeks. +: p<0.05 vs corresponding sham.

### 2. Effect of RAS blockade and alternative antihypertensive treatment on interstitial myocardial fibrosis and capillarisation in SNX (table 2, *figs. 2B*,*3*,*4*)

#### Enalapril (E), but not furosemide/dihydralazine (F/D) treatment caused regression of myocardial fibrosis in SNX animals (fig. 2B,3)

**Figure 3:**
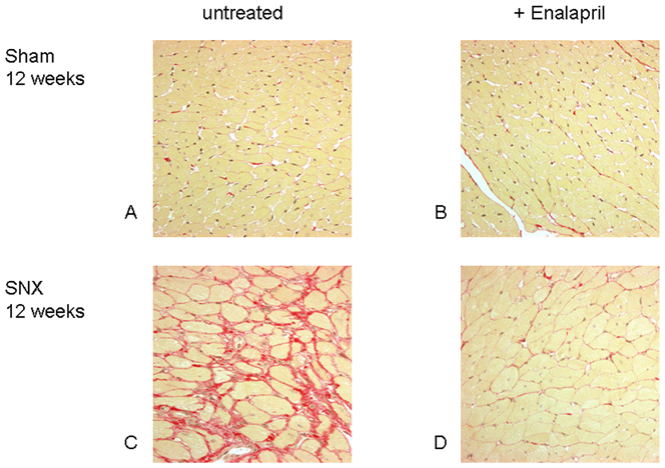
Myocardial fibrosis in untreated sham operated animals (A), sham+enalapril (B), untreated SNX 12 weeks (C) and SNX + enalapril (D). Note increased myocardial fibrous tissue content (depicted in red) in untreated SNX at 12 weeks (C) compared to untreated and treated sham (A,B). Complete regression of interstitial fibrosis is seen at 12 weeks after 4 weeks treatment with enalapril (D).Sirius red stain, magnification x 400.

At weeks 8 and 12 volume density of interstitial tissue (Vv int in %) as an index of myocardial fibrosis was significantly higher in untreated SNX than in sham operated rats (fig. 2B,3). Whereas RAS blockade with E significantly lowered the percentage of myocardial fibrous tissue compared to the values of untreated SNX at weeks 8 and 12, alternative antihypertensive treatment with F/D did not show such an effect.

**Figure 4:**
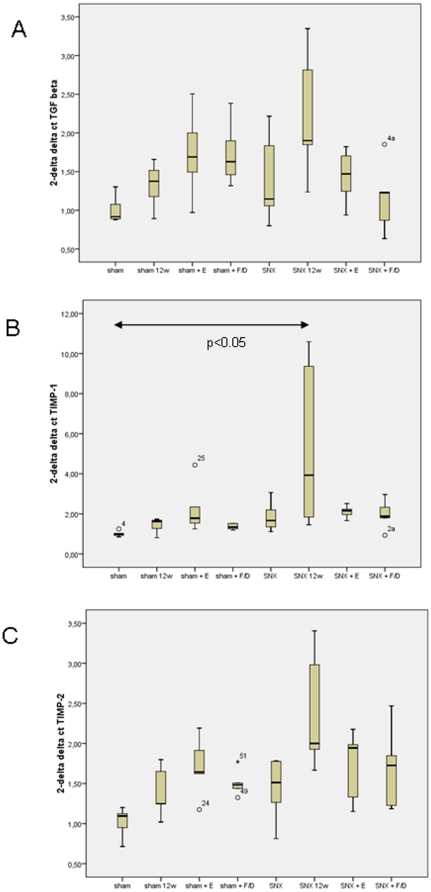
Effect of treatment with the ACE-I enalapril or furosemide/dihydralazine on cardiac mRNA expression of TGF-β (A), TIMP-1 (B) and TIMP-2 (B). Increased TGF-β mRNA expression in untreated SNX was lowered by both antihypertensive treatments. Cardiac TIMP-1 gene expression was also significantly higher in untreated SNX 12 weeks than in sham and SNX 8 weeks; RAS blockade by ACE-I and alternative antihypertensive treatment both lowered cardiac TIMP-1 gene expression in SNX animals. The same tendency was seen for TIMP-2 mRNA expression. The data are provided as box plots of the ΔCT analysis. ° indicate outlyers.

**Table 2:** Effect of enalapril (E) or furosemide/dihydralazine (F/D) treatment from week 8–12 in sham-op and SNX on intramyocardial arterioles and capillaries.

group	lumen diameter [µm]	wall thickness [µm]	wall:lumen ratio [µm/µm*10^−2^]	Lv [mm/mm^3^]	Intercapillary distance [µm]
**SHAM 8 wks** **n = 10**	30.0±4.9	3.3±0.5	12.4±2.7	4139±486	16.8±1.1
**SHAM 12 wks** **n = 10**	25.9±2.7	4.3±1.0	17.7±4.7	5023±624	15.3±1.0 **b**
**SHAM +E** **n = 11**	27.9±6.9	4.8±2.0	18.8±10.4	5339±915	14.9±1.2 **b**
**SHAM + F/D** **n = 8**	33.5±4.8	2.9±0.4	10.7±4.4	4641±429	15.7±0.7
**SNX 8 wks** **n = 8**	32.3±5.4 **c,d**	4.1±0.6 **c,d**	13.9±4.1 **c,d**	4086±517	16.9±1.1
**SNX 12 wks** **n = 8**	26.9±2.7 **b**	5.4±0.7 **b**	21.8±3.2 **b**	4532±355 **d**	16.0±0.7 **d**
**SNX +E** **n = 11**	25.2±4.3 **b**	5.9±1.7 **a,b**	24.7±8.8 **a,b**	3908±486 **a,c**	17.3±1.2 **a,c**
**SNX + F/D** **n = 7**	32.1±4.4 **d**	3.7±0.6 **c,d**	12.9±3.2 **c,d**	4610±553 **d**	15.9±1.0 **d**
**analysis of variance**	p<0.05	p<0.05	p<0.05	p<0.05	p<0.05

Mean ± standard deviation.

a) p<0.05 vs. corresponding SHAM.

b) p<0.05 vs. SNX 8 wks.

c) p<0.05 vs. SNX 12 wks.

d) p<0.05 vs. SNX+E.

#### The effect of Enalapril (E) on cardiac fibrosis in SNX animals was only partly dependent on lowering of TGF-β and TIMP expression (fig. 4)

In the hearts of untreated SNX at 12 weeks markedly increased TGF-β, TIMP-1 and TIMP-2 mRNA expression compared to control animals was found by TaqMan PCR (fig. 4). Due to the high standard deviation of cardiac gene expression the difference was only statistically significant for TIMP-1 (fig. 4B). Expression of all 3 profibrotic genes was again markedly lowered by both antihypertensive treatments, but the differences were not statistically significant (fig. 4).

#### Enalapril (E) and furosemide/dihydralazine (F/D) treatment had no beneficial effect on reduced myocardial capillary density in SNX (table 2)

At 8 weeks after SNX capillary length density (Lv), i.e. the total length of capillaries per unit myocardial volume, as a three-dimensional parameter of myocardial capillary supply was comparable in untreated SNX and sham animals. After 12 weeks myocardial capillary length density was markedly lower in untreated SNX compared to sham. Because of the high standard deviation this marked difference failed to be statistically significant. Antihypertensive treatment with either E or F/D did not improve myocardial capillary density in either SNX or sham animals. In SNX+E animals Lv was even lower compared to untreated SNX and F/D treatment. Changes in intercapillary distance, an important parameter of myocardial blood supply, went in parallel. In addition, myocardial intercapillary distance was significant higher in SNX 8 weeks than in sham (table 2).

### 3. Effect of RAS blockade and alternative antihypertensive treatment on changes of intramyocardial arterioles

#### Treatment with furosemide/dihydralazine (F/D), but not with Enalapril reversed wall thickening of intramyocardial arterioles in SNX (table 2)

All intramyocardial arterioles with lumen diameters between 15 and 50 µm were measured. The mean number of intramyocardial arterioles assessed per animal ranged from 6 to 32. The cumulative frequencies of arteries were not different between the groups excluding a sampling error. This conclusion is further supported by the fact that the mean lumen diameter was not significantly different between untreated sham and SNX 8 weeks (table 2). Lumen diameter in SNX 12 weeks and SNX+E was significantly lower than in SNX 8 weeks. In addition, it was significantly higher in SNX+F/D than in SNX+E. Wall thickness, wall:lumen ratio and media area (not shown) of intramyocardial arterioles were significantly higher in SNX 12 weeks and SNX+E than in SNX 8 weeks. Interestingly, values were significantly lower in the SNX+F/D group.

### 4. Effect of RAS blockade and alternative antihypertensive treatment on changes of the aortic wall

#### Enalapril (E), but not furosemide/dihydralazine (F/D) lowered increased aortic wall thickness in SNX (table 3, fig. 5)

Aortic lumen diameter and lumen area were not significantly different between untreated sham and untreated SNX at 8 and 12 weeks. Lumen diameter was significantly lower in SNX+E compared to sham+E and significantly higher in SNX+F/D than in SNX 8 weeks presumably indicating vessel dilatation. In contrast, aortic media thickness at week 8 was significantly higher in SNX than in sham, whereas at week 12 due to the somewhat higher standard deviation there was only a tendency to higher values in SNX. Treatment of SNX with E, but not with F/D lowered aortic media thickness (table 3).

**Figure 5:**
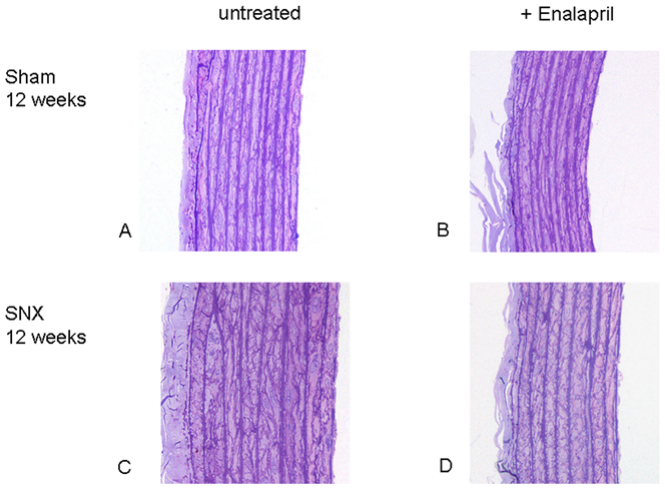
Effect of treatment with the ACE-I enalapril (E) on aortic wall thickness and aortic remodelling in sham (A,B) and SNX rats (C,D). The increase in aortic wall thickness in untreated SNX (C) compared to untreated and E-treated sham (A,B) reversed by antihypertensive treatment with enalapril (D).

**Table 3:** Effect of enalapril (E) or furosemide/dihydralazine (F/D) treatment from week 8–12 in sham-op and SNX on the aortic wall.

group	lumen diameter [µm]	lumen area [mm^2^]	media thickness [µm]	media area [mm^2^]	number of VSMC per media area [1/mm^2^]
**SHAM 8 wks** **n = 10**	1177±375	1.19±0.71	88.9±8.9	0.422±0.17	19.2±7.07
**SHAM 12 wks** **n = 10**	1487±464	1.89±0.85	97.6±11.7	0.658±0.25	18.4±2.60
**SHAM +E** **n = 11**	1109±395	1.08±0.78	83.2±7.32	0.431±0.12	18.3±2.27
**SHAM + F/D** **n = 8**	1809±294	2.63±0.77	100±11.9	0.705±0.21	20.4±2.94
**SNX 8 wks** **n = 8**	1312±383	1.45±0.82	105±6.47 **a**	0.582±0.20	14.4±2.53 **a**
**SNX 12 wks** **n = 8**	1676±298	2.27±0.81	109±21.9	0.792±0.30	10.8±2.36 **a.d**
**SNX + E** **n = 11**	1471±270 **a**	1.75±0.59	98.6±18.4 **a**	0.634±0.21 **a**	14.8±2.00 **a.c**
**SNX + F/D** **n = 7**	1854±530 **b**	2.89±1.26 **b.d**	109±19.7	0.859±0.25 **b.d**	15.4±2.22 **a.c**
**analysis of variance**	p<0.05	p<0.05	p<0.05	p<0.05	p<0.05

Mean ± standard deviation.

a) p<0.05 vs. corresponding SHAM.

b) p<0.05 vs. SNX 8 wks.

c) p<0.05 vs. SNX 12 wks.

d) p<0.05 vs. SNX+E.

#### Enalapril (E) and furosemide/dihydralazine (F/D) improved aortic VSMC/matrix ratio in SNX animals (table 3, fig. 5)

At weeks 8 and 12 the number of aortic VSMC per unit media area was significantly lower in untreated SNX compared to sham (table 3). In parallel, aortic extracellular matrix content as seen in fibrous tissue stains and semithin sections (fig. 5) was higher in untreated SNX (fig. 5C) than in sham (fig. 5A) indicating structural remodelling of the aortic wall. Of note, in both treated SNX groups (SNX+E, SNX+F/D) the number of VSMC per aortic media area was significantly increased compared to untreated SNX (tab. 3), but there was no effect on elastic fibre content (data not shown).

## Discussion

In the present study the effect of 4 weeks of ACE inhibition (ACE-I) with high-dose enalapril (E) treatment on the regression of LVH and accompanying abnormalities of myocardium and aorta were investigated in an experimental modelof chronic renal failure, i.e. the subtotally nephrectomized rats (SNX). Potential effects of blood pressure (bp) lowering by E were controlled for by a treatment arm with comparable bp lowering, i.e. a combination of furosemide and dihydralazine (F/D). Treatment with E, but not with F/D led to regression of LVH and myocardial interstitial fibrosis. In contrast, no beneficial effect of E was seen on reduction of myocardial capillary density, increased intercapillary distance or thickening of intramyocardial arteries in SNX, respectively.

Thickening of the aortic media in SNX was only partly, but not completely regressed by E treatment. The structural alterations of aortic media in SNX, i.e. decreased ratio VSMC:extracellular matrix were positively affected by both antihypertensive treatments.

Some methodological aspects of the present study deserve further comments:

In our hands the standard model of SNX induces reproducibly stable moderate chronic renal failure that is accompanied by only moderately increased systolic bp [5], [16]. This is in contrast to findings in alternative models of renal insufficiency, i.e. the renal artery ligation model where bp is markedly increased [29]. In the SNX model by surgical ablation plasma Ang II is decreased, presumably due to volume overload, while increased local formation of Ang II has been reported in extrarenal resistance vessels [30]. The mechanisms contributing to higher local Ang II levels may include higher levels of the precursor protein angiotensinogen, and a decreased degradation of Ang II [30]. We are not aware of studies directly addressing such mechanisms in the heart. However, an upregulation of Ang II AT1 receptors and increased local renin mRNA in the myocardium has been reported after subtotal nephrectomy [7], [13], [31]. Higher AT1 receptor density may lead to a more pronounced local cardiac effect of Ang II. Of note, in a recent study [32] renal AT1 receptors were found to be required for the development of Ang II-dependent hypertension and cardiac hypertrophy suggesting that the major mechanism of action of RAS inhibitors in hypertension is attenuation of Ang II effects in the kidney.

In previous studies our group had shown that treatment with the ACE-I ramipril prevented the development of LVH and myocardial fibrosis in SNX rats [4], [16]. When designing the present study we reasoned that in contrast to prevention [33], regression of already altered heart morphology might require a higher dose of E, e.g. 48 mg/kg body weight [22] similar to the high doses necessary to cause regression of glomerular sclerosis [23]. In addition, there is accumulating data in the proteinuric nephropathy setting that using super-high doses of AT1 blockers can indeed be of added clinical benefit [34]. The combination of furosemide and dihydralazine was used to achieve comparable bp control; previous studies in this laboratory had documented that this combination did not affect morphological changes of the heart in SNX [5]. This is also in line with our past observation that the development of LVH in SNX is bp independent [4], [10], [11] since it cannot be prevented by bp lowering with either calcium channel blockers or other bp lowering agents, but only with ACE-I, endothelin receptor blockade or sympatholytic agents. These observations point so some pathogenetic involvement of these and other systems like for example increased PTH [1]–[3].

As already mentioned [32] Ang II was shown to affect hypertension and subsequent heart hypertrophy through its AT1 receptors in the kidney. In the absence of hypertension (due to the renal knockout of AT1), cardial AT1 receptors were not sufficient to cause hypertrophy. At first glance, these findings appear to conflict with the notion that the effects of RAS blockade were independent from bp in our model. However, several aspects of the experimental setup were different. Apart from species differences and different strategies to interfere with the RAS, kidney AT1 receptors were not knocked out in our model but some degree of Ang II signaling through AT1 was certainly present in the kidney. Further, the effects of local RAS activation on the heart may be more pronounced in the presence of volume overload which could occur in subtotal nephrectomy. We have to acknowledge, however, that some of the effects of F/D treatment can be presumably explained by differences in bp before the start of treatment and particulary by increased natriuresis which has been shown to help to prevent and regress LVH [19], [20].

There is good evidence for a role of the local cardiac RAS (and possibly bradykinin) in the pathogenesis of myocardial fibrosis [35]. Gonzalez et al. [36] showed that AngII signalling via the AT-1 receptor, MAPK/ERK and SMAD stimulates procollagen I formation and inhibits the activity of collagenases resulting in increased collagen I accumulation. This cascade is further modulated by TGF-β, PDGF, aldosterone, integrins and PAI-1 [36]. Of note, in the present experimental study TGF-β, TIMP-1 and TIMP-2 mRNA expression was not specifically altered by E treatment (and this was also confirmed for TGF-β on the protein level using immunohistochemistry, data not shown). In experimental renal failure as well, ACE-I therapy attenuated the development of myocardial fibrosis and cardiomyocyte hypertrophy as a result of activating lysosomal proteinases [37]. In past experiments we treated SNX animals with a combination of ACE-I and bradykinin antagonist; thereby the bradykinin antagonist abrogated the beneficial effect of ACE-I [35]. In addition to inhibiting cardiac fibrosis, ACE-I also reduced the loss of cardiomyocytes by apoptosis in SNX rats [4].

Apart from activation of the local RAS in the heart, changed elasticity of central arteries, mainly the aorta, also contribute to the development of LVH in CKD. This was documented by London et al in CKD patients [38]. Reduced aortic elasticity increases the pressure load imposed upon the left ventricle thus aggravating LVH. It is therefore of note that in the present study we confirmed reduced elastic fibre content and disturbed architecture of elastic fibres in the aorta of SNX animals [5], [16]. This will increase aortic stiffness together with the observed reduced VSMC numbers and increased extracellular matrix. Of note, on a qualitative base these structural alterations were positively affected by high-dose ACE-I. This is in line with data on a protective effect of ramipril in animal models of hypertension without renal dysfunction [39].

Our previous studies had documented a selective and marked increase of myocardial interstitial non-vascular tissue in SNX rats [3] and in uremic patients [40]. In uremic patients myocardial fibrosis was shown to be independent of potential confounders such as hypertension, diabetes or duration of dialysis [41]. In SNX animals treatment with the ACE-I ramipril, but not with the sympatholytic agent moxonidine or the calcium channel blocker nifedipine prevented the development of myocardial fibrosis [4]. This finding is confirmed and extended by the results of the present study: Enalapril, but not alternative antihypertensive treatment with furosemide/dihydralazine caused even regression of established myocardial fibrosis. Regression is proven by the observation that at week 12 the interstitial tissue area of SNX animals which had received high dose E for 4 weeks was even lower than the baseline value in untreated SNX at week 8. Apart from lowering of AngII by ACE-I a complementary explanation might be accumulation of bradykinin and we had already provided evidence for this in an earlier study using the SNX model [35]. Minshall et al. [42] documented bradykinin-2-receptors on neonatal and adult rat fibroblasts as well as cardiomyocytes. Stimulation of these receptors influences proliferation and protein synthesis directly and indirectly (via nitric oxide or prostacyclin) in various tissues [43]. It is therefore conceivable that bradykinin accumulation in the myocardium inhibits collagen synthesis by interstitial fibroblasts [44]. This idea is in line with increased PCNA positivity of interstitial cells indicating activation of fibroblasts and increased TGF-β expression in the heart of SNX animals compared to sham operated controls [10]. Of note, in renal failure PTH has a permissive role on the activation of interstitial fibroblasts in vitro and in vitro [3]. Ultrastructural analysis in SNX animals 14 days after subtotal nephrectomy documented early selective activation of cardiac interstitial fibroblasts, but not of endothelial cells [2]. Furthermore, Suzuki et al. documented that the increase in interstitial matrix is due to both increased production by activated fibroblasts and decreased matrix removal by MMPs [37].

The length density of intramyocardial capillaries as a three-dimensional parameter of myocardial capillary supply is significantly reduced in experimental renal failure^8^ as well as in uremic patients [40]. Reduced myocardial capillary supply increases the intercapillary distance thus lowering pO2 midways between the capillaries thus rendering the myocardium more susceptible for ischemic injury [8], [10]. Deficient capillary supply is a feature of LVH in renal failure. Its development is independent of bp [16]. In SNX reduced myocardial capillary supply is prevented by selective blockade of the sympathetic and endothelin systems [16], [45] as well as by the antioxidant vitamin E [46] but not by ACE-I [16]. The present study extends the latter finding by documenting that low capillary density is not reversed by E either; we noted even a tendency to lower values after E treatment. This finding could be due to a blockade of the promitogenic effect of Ang II on endothelial cells [47] by ACE-I. An alternative explantation may be exhaustion of the endothelial cell pool after intense endothelial cell/mesenchymal transition [48]. As expected based on the findings of the various prevention studies treatment with F/D did not show any beneficial effect on reduced myocardial capillary density.

Thickening of intramyocardial arterioles as well as of extracardiac arteries and veins is found in SNX rats and in CKD patients [5], [10], [16]. In the present study we noted only a tendency to higher wall thickness in SNX at 8 weeks compared to sham animals which increased with the duration of renal failure and was significant at 12 weeks. This increase in wall thickness was not regressed or prevented by E which is in line with previous studies of our group [5], [16], but contrasts to data of Kakinuma et al [49] who described a protective effect of ACE-I with respect to vascular thickening in experimental renal failure. The marked effect of F/D on intramyocaridal arteriolar wall thickness and wall: lumen ratio was unexpected. F/D treatment had also an effect on the arteriolar diameter which is increased possibly indicating arteriolar dilatation.

In summary, in subtotally nephrectomized rats, high doses of the ACE-I enalapril cause regression of LVH and interstitial myocardial fibrosis. In addition, regression of abnormal aortic wall texture is observed. These results of this short term study extend previous experimental findings that lower doses of ACE-I prevent cardiac and aortic pathology and provide another argument for the clinical use of ACE-I in patients with CKD and established cardiovascular pathology. In remarkable contrast, myocardial capillary density and intercapillary distance, crucial determinants of tissue hypoxia tolerance, were not positively affected by ACE-I treatment. It remains to be investigated, however, whether longer treatment periods or addition of combination treatment with AT2 or aldosterone receptor blockers or renin inhibition, respectively, may increase the effects.
